# Synthesis of [^211^At]4-astato-L-phenylalanine by dihydroxyboryl-astatine substitution reaction in aqueous solution

**DOI:** 10.1038/s41598-021-92476-6

**Published:** 2021-06-21

**Authors:** Yoshifumi Shirakami, Tadashi Watabe, Honoka Obata, Kazuko Kaneda, Kazuhiro Ooe, Yuwei Liu, Takahiro Teramoto, Atsushi Toyoshima, Atsushi Shinohara, Eku Shimosegawa, Jun Hatazawa, Koichi Fukase

**Affiliations:** 1 Institute for Radiation Sciences, Osaka University, Suita, 565-0871 Japan; 2 Research Center for Nuclear Physics, Osaka University, Suita, 565-0871 Japan; 3 Department of Tracer Kinetics and Nuclear Medicine, Graduate School of Medicine, Osaka University, Suita, 565-0871 Japan; 4 Department of Chemistry, Graduate School of Science, Osaka University, Toyonaka, 560-0043 Japan; 5 Department of Molecular Imaging in Medicine, Osaka University Graduate School of Medicine, Suita, 565-0871 Japan

**Keywords:** Drug discovery, Chemistry

## Abstract

Astatine-211 (^211^At)-labeled phenylalanine is expected to be a promising agent for targeted alpha-particle therapy for the treatment of patients with glioma. The existing reactions to prepare the labeled compound usually require organic solvents and metals that are toxic and hazardous to the environment. In this study, we developed a novel method wherein astatination was realized via the substitution of ^211^At for a dihydroxyboryl group coupled to phenylalanine. [^211^At]4-astato-L-phenylalanine was obtained as the carrier-free product in aqueous medium in high radiochemical yields (98.1 ± 1.9%, n = 5). The crude reaction mixture was purified by solid-phase extraction, and the radiochemical purity of the product was 99.3 ± 0.7% (n = 5). The high yield and purity were attributed to the formation of [^211^At]AtI and AtI_2_^−^ as the reactive intermediates in the astatination reaction. The reaction did not require any organic solvents or toxic reagents, suggesting that this method is suitable for clinical applications.

## Introduction

Radiopharmaceutical therapy, including targeted alpha-particle therapy (TAT), has recently emerged as a novel therapeutic modality for the treatment of tumor^1^. We previously demonstrated the utility of [^211^At]4-astato-L-phenylalanine ([^211^At]APA) for the treatment of glioma in tumor-bearing mice by means of TAT^2^. [^211^At]APA specifically accumulates in tumor cells and are transported by the LAT1 transporters, which are predominantly expressed on the surface of various tumor cells including the glioma cells. In a mouse model xenografted with rat glioma cells, [^211^At]APA significantly decreased the tumor volume after a single injection of the agent (dose range: 0.1–1.0 MBq/mouse) in the mouse.

Phenylalanine has been previously labeled with ^211^At using different methods^3–5^. However, most of these methods have certain limitations. The existing reactions for the synthesis of radiolabeled compounds either require the use of toxic and hazardous chemicals or are unable to yield a carrier-free final product. For instance, Meyer et al. synthesized [^211^At]APA via the nucleophilic halogen exchange reaction by heating the corresponding bromo- and iodo-derivatives in the presence of copper sulfate as a catalyst at 140 ℃ for 60 min^3,4^. However, the bromo- and iodo-derivatives could not be removed in the final product. They also prepared [^211^At]APA by the electrophilic destannylation of *N*-Boc-4-tributylstannyl-L-phenylalanine; however, the organic tin compound used in this reaction is hazardous for the environment. Watanabe et al. recently reported the synthesis of [^211^At]APA via electrophilic desilation; the corresponding ^211^At-labeled triethylsilyl substituted precursor was heated at 70 ℃ for 10 min in a methanol–trifluoroacetate solvent mixture^5^. The overall radiochemical yield in this method was in the range 65–85%. However, the use of toxic reagents and hazardous solvents such as organic tin, methanol, trifluoroacetate, and chloroform must be avoided, in order to comply with the International Council of Harmonization of Technical Requirements for Pharmaceuticals for Human Use guidelines, good manufacturing practice, and other laws. Yan et al. developed a copper-catalyzed transformation of aryl iodides from arylboronic acid at room temperature via the reaction with potassium iodide in water as an environmentally friendly solvent^6^. Reilly et al. employed the copper-catalyzed transformation for the ^211^At-astatination of arylboronic esters as well as for its iodination^7^. They prepared an [^211^At] PARP inhibitor in high radiochemical yields by using the corresponding boronic ester precursor and [^211^At]NaAt in the presence of Cu(pyridine)_4_OTf in a 4:1 mixture of methanol and acetonitrile. This method could be successfully applied for the astatination of antibodies and small molecules^8^.

In this study, we aimed to develop a new and improved method for the preparation of [^211^At]APA based on our previous method using 4-borono-L-phenylalanine (BPA) as the starting molecule. [^211^At]APA was synthesized by the electrophilic substitution of ^211^At for a dihydroxyboryl (or borono) group on an aromatic ring of the corresponding precursor molecule BPA using *N*-bromosuccinimde (NBS; NBS method) as an oxidant or KI (KI method). The radiochemical yield (RCY) and radiochemical purity (RCP) were better with the latter method. The reagents and compounds used for the synthesis of [^211^At]APA were commercially approved drugs and physiologically relevant for clinical use. The entire synthesis could be accomplished in aqueous media; no organic solvents or toxic metals were required, suggesting that this method is relevant for practical applications. We also elucidated the differences between the chemical properties of iodine and astatine for the labeling reactions.

### Results

**Figure 1:**
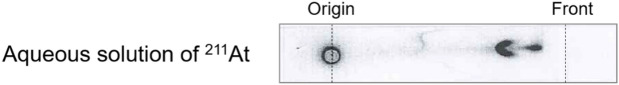
Representative radio-TLC profile of the aqueous solution of ^211^At.

^211^At separated and purified after dry-distillation is generally dissolved and recovered in organic solvents, such as chloroform, methanol, and ethanol^5,9–11^. Chloroform seems to be the most efficient solvent for collecting ^211^At with high recovery yields. In chloroform, however, unknown forms of the ^211^At species have been detected, some of which are probably generated from the radiolysis of chloroform, as suggested by Aneheim et al.^9^. In methanol too, ^211^At is present in the form of an unknown species At (1) just after the dry-distillation, which later degrades to another species, At (2), in a radiation dose-dependent manner^12^. As an alternative to organic solvents, we choose Water for Injection (WFI) for the dissolution of ^211^At trapped in the Teflon tube cooled by ice-water after the dry-distillation^13^. The recovery yields of ^211^At in WFI were in the range 40–80%. A representative radio-thin layer chromatography (TLC) profile of the aqueous solution of ^211^At is shown in Fig. 1. There were three or more unknown ^211^At species in the aqueous solution. The radioactivity profiles during TLC of the aqueous solution of ^211^At varied from batch to batch, probably due to the slight differences in the oxidation conditions among the batches (Supplemental Fig. S1). A portion of the ^211^At solution was volatile, while the other portion stuck to the Teflon tube. We used the aqueous solutions of ^211^At containing the different ^211^At species without any further treatment for the subsequent radiolabeling experiments.

**Figure 2:**
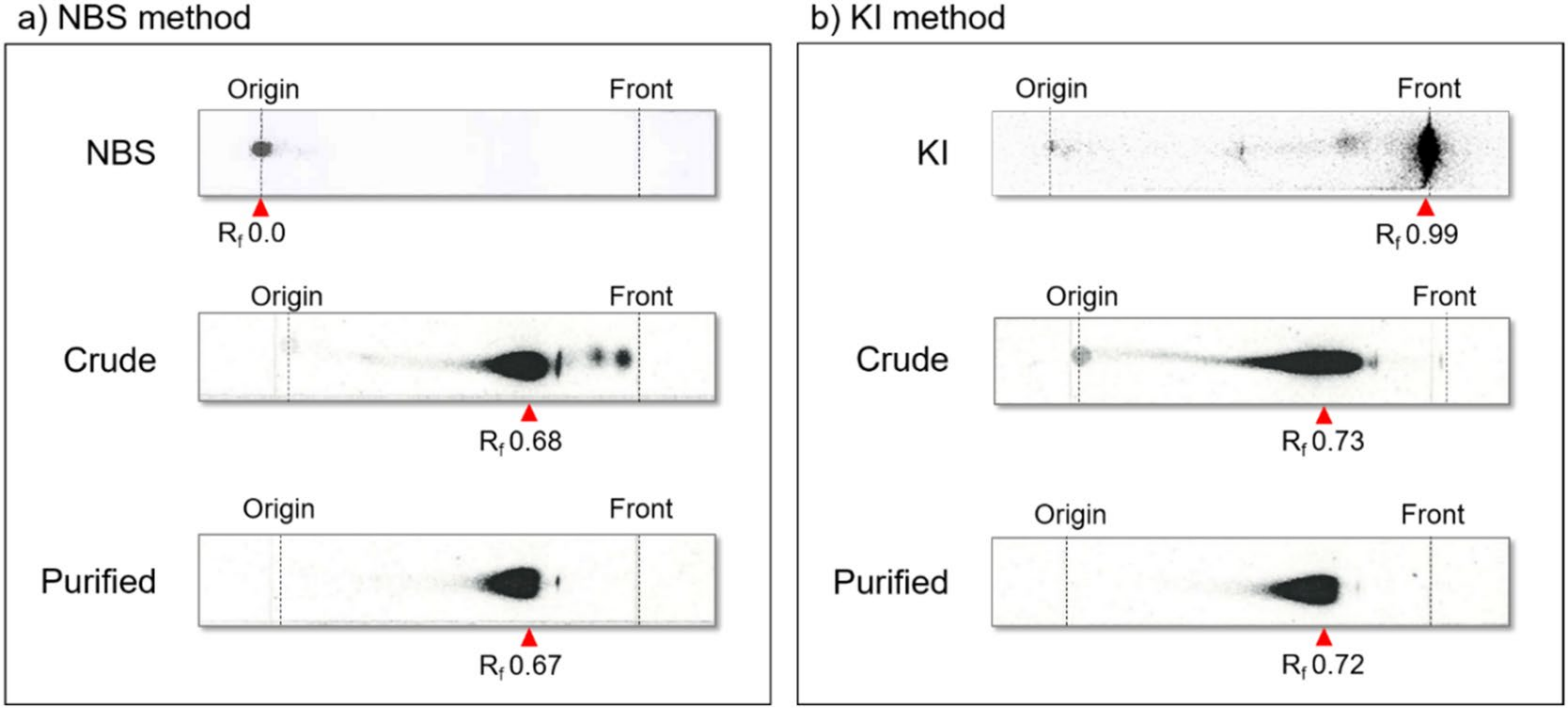
Radio-TLC analyses of ^211^At-labeled intermediates and [^211^At]APA. (**a**) NBS method: Reaction mixture after addition of NBS in the aqueous solution of ^211^At (top); crude reaction mixture reacted with BPA, followed by the addition of ascorbic acid (middle); after SPE purification (bottom). (**b**) KI method: Reaction mixture after addition of KI solution in the aqueous solution of ^211^At (top); crude reaction mixture reacted with BPA (middle); after SPE purification (bottom).

The radio-synthesis of [^211^At]APA was conducted with the aqueous solution of ^211^At using either NBS or KI. When NBS solution was added to the aqueous solution of ^211^At, only a single radioactive spot (R_f_ 0.0) was detected as an intermediate; this could be attributed to the formation of [^211^At]*N*-astatosuccinimide (Fig. 2a, top). The intermediate was reacted with BPA in an aqueous medium at room temperature for 30 min, followed by the addition of ascorbic acid. Consequently, [^211^At]APA (R_f_ 0.68) was obtained as the crude product (Fig. 2a, middle). A few minor radioactive impurities were detected at R_f_ 0.8–1.0, probably corresponding to inorganic ^211^At species including the astatide ions. The impurities were removed upon solid-phase extraction (SPE) purification of the crude product using a HLB cartridge, yielding purified [^211^At]APA (R_f_ 0.67) (Fig. 2a, bottom).

When KI solution was added to the aqueous solution of ^211^At, a prominent radioactive spot corresponding to an intermediate was detected in the solvent front (R_f_ 0.95–1.0) (Fig. 2b, top). The intermediate was reacted with BPA in an aqueous medium at room temperature for 30 min, resulting in the formation of [^211^At]APA (R_f_ 0.73) as the crude product (Fig. 2b, middle). Purified [^211^At]APA (R_f_ 0.72) was obtained after the SPE purification (Fig. 2b, bottom).

The RCYs of [^211^At]APA in the crude reaction mixture obtained using NBS and KI were 90.8 ± 2.7% (n = 6) and 98.1 ± 1.9% (n = 8), respectively. The RCPs of the products one hour after the SPE purification were 96.3 ± 2.0% (n = 5) and 99.3 ± 0.7% (n = 5), respectively. Both the purified products were stable for 24 h (RCP > 95%).

**Figure 3:**
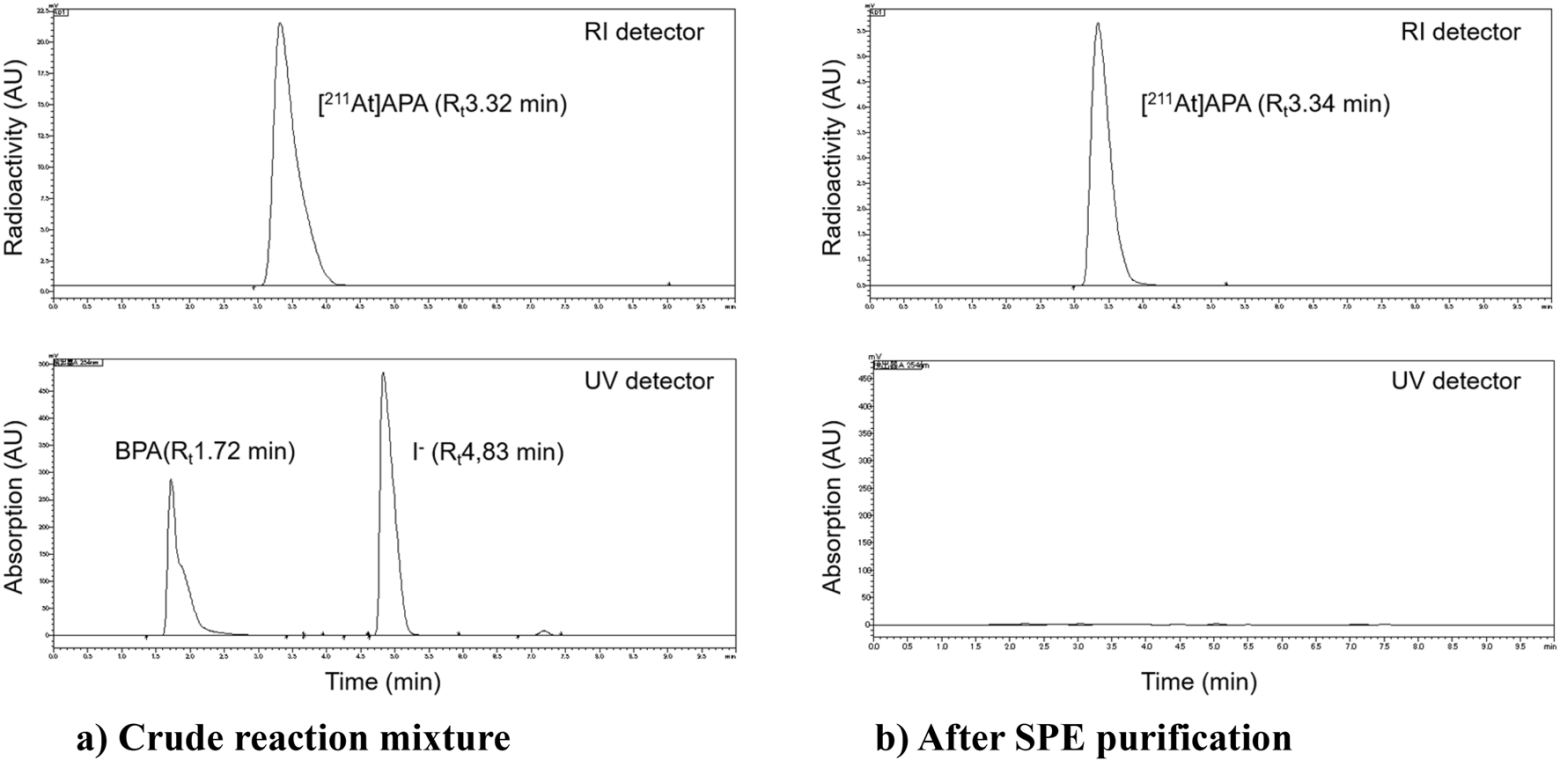
HPLC profiles of [^211^At]APA synthesized by the KI method. (**a**) Crude reaction mixture, (**b**) after SPE purification. Top panel: Radioactive trace; Bottom panel: UV trace.

We next analyzed [^211^At]APA synthesized by the KI method using reversed-phase ion-pair high-performance liquid chromatography (HPLC; Fig. 3). The major radioactive peaks appeared at R_t_ 3.32 min for the crude reaction mixture (Fig. 3a) and at 3.34 min for the solution obtained after SPE purification (Fig. 3b). These peaks were speculated to correspond to [^211^At]APA, which was eluted ~ 0.5 min after the elution of the corresponding iodinated compound 4-iodo-L-phenylalanine (IPA; R_t_ 2.82 min, Supplemental Fig. S2). ^211^At-astatide ions (R_t_ 7.0–7.4 min) was not detected in any of the solutions. Nonradioactive chemicals BPA (R_t_ 1.72 min) and iodide (R_t_ 4.83 min) were observed in the crude reaction mixture upon UV detection at 254 nm. Masses of BPA and iodide were reduced to around one tenth or less after the SPE purification of the crude reaction mixture. IPA was not detected in the crude reaction mixture or in the SPE-purified solution, suggesting that iodide ions are not involved in the substitution reaction with BPA under the employed reaction conditions. This was also confirmed by the HPLC analysis of the mixture of BPA and KI (Supplemental Fig. S3).

The corresponding radioactive and nonradioactive iodinated compounds were synthesized in a similar manner using the NBS method. The stable isotope of IPA was prepared using NaI, which was reacted with chemically equivalent amounts of BPA and NBS. The synthesized product and the authentic compound (IPA) were analyzed by chiral liquid chromatography-mass spectrometry (LC–MS) (Supplemental Fig. S4). The retention time of the chemically synthesized molecule was 3.13 min and the extraction mass (+ H) was 291.9835 Da (estimated chemical formula is C_9_H_10_INO_2_, and m/z calculated for C_9_H_11_INO_2_ [M + H]^+^ is 291.9834). The retention time and extraction mass agreed with those of authentic IPA (molecular mass: 290.9756 Da). IPA was stoichiometrically synthesized, and no bromo-substituted by-products were detected in the reaction mixture.

**Figure 4:**
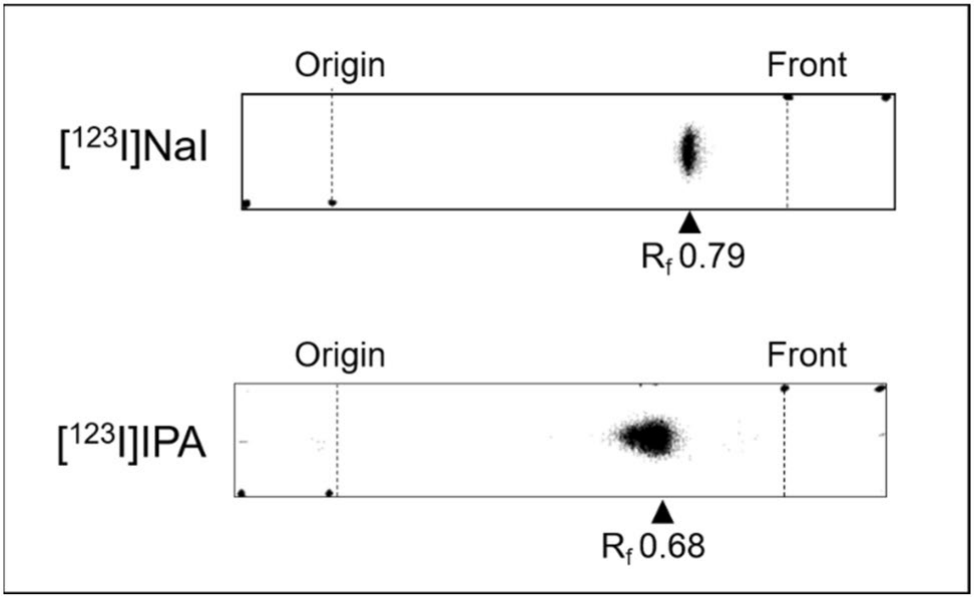
TLC profiles of [^123^I]NaI and [^123^I]IPA synthesized by the NBS method.

[^123^I]IPA was synthesized by the reaction of iodine-123-labeled sodium iodide ([^123^I]NaI) with BPA using the NBS method under the same conditions as those for the synthesis of [^211^At]APA. [^123^I]NaI was detected as a single radioactive spot at R_f_ 0.79 on the TLC plate (Fig. 4, top). The radioactive product in the reaction mixture was identified to be [^123^I]IPA (R_f_ 0.68, RCP = 92.0%), as the R_f_ value was similar to that of authentic IPA estimated from TLC (Fig. 4, bottom).

### Discussion

One of the most important objectives of this study was to investigate the differences and similarities in the chemical reactivities of iodine and astatine. Considering that the radio-halogenation was conducted in the last step of the synthesis, the reagents, solvents, and by-products used should be non-toxic. We choose dihydroxyboryl as the leaving group of the precursor molecule, which was halogenated by the replacement of the dihydroxyboryl group with ^211^At and ^123^I. The dihydroxyboryl-substituted molecules, such as BPA, are soluble in water since the dihydroxyboryl groups are hydrophilic; however, BPA is insoluble in most of the organic solvents. The fact that the dihydroxyboryl group is released from the precursor molecule after the substitution reaction in an aqueous solution, without the involvement of any toxic reagent, is clinically relevant. The 7% (w/v) aqueous solution of sodium hydrogen carbonate, which is commercially available around the globe as a drug for intravenously injectable solutions, was used for the dissolution of BPA. We found that the drug is an excellent solvent for the dissolution of dihydroxyboryl-substituted molecules as well as BPA. The drug is weakly basic, with the pH varying from 7.0 to 8.5. This is suitable for both astatination and iodination of BPA. Our findings suggest that deboronation is one of the most efficient routes for the astatination of aryl compounds and antibodies, as concluded in the previous studies conducted using copper catalysts^6–8^.

#### Mechanism of radio-halogenation

**Figure 5:**
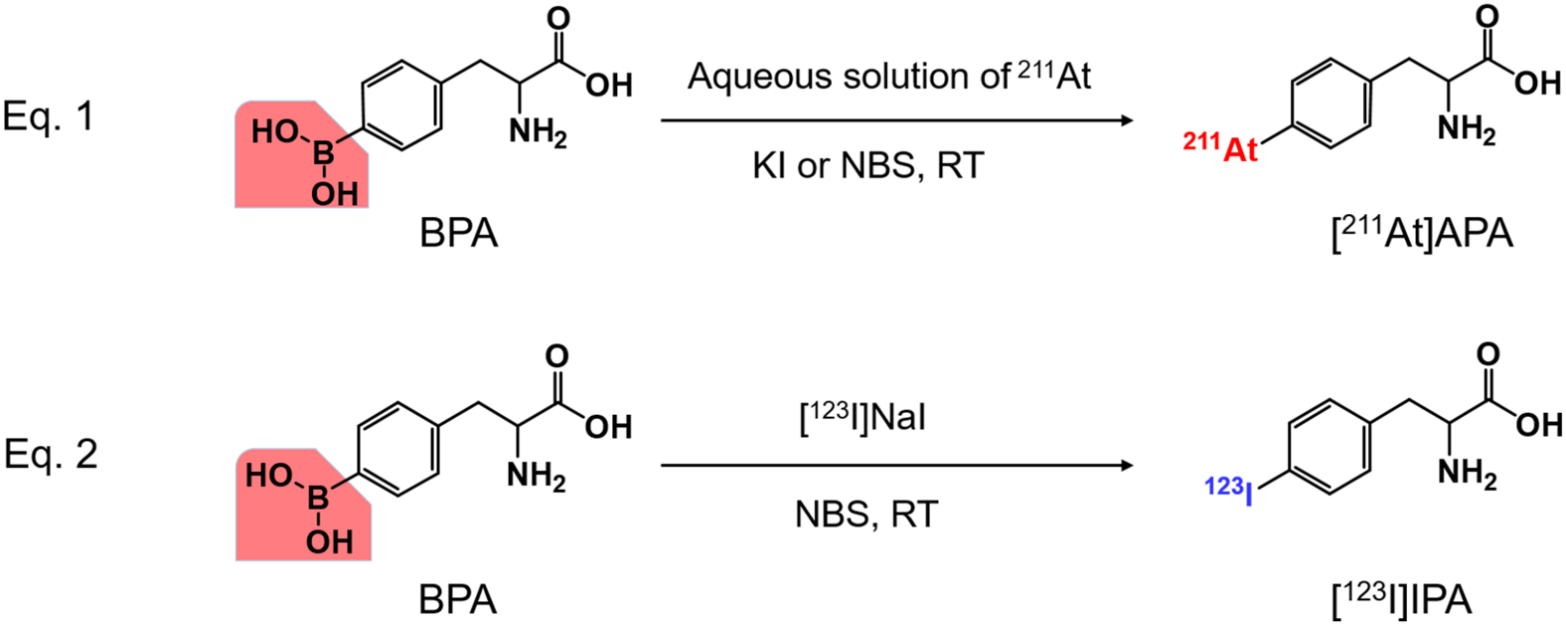
Radio-halogenation of BPA by halogen-dihydroxyboryl substitution reaction.

The electrophilic substitution reaction of BPA with ^211^At was realized by both the NBS method and the KI method (Fig. 5, Eq. 1), whereas the iodination proceeded by the NBS method but not by the KI method (Fig. 5, Eq. 2). Interestingly, the radio-astatination of BPA with ^211^At occurred directly and immediately after the addition of the aqueous solution of ^211^At in the BPA solution, in the absence of NBS or KI; the RCY of [^211^At]APA was 50% or higher. This indicated that the major chemical forms of ^211^At in the aqueous solution are some of the oxidized species that are in the monovalent (^211^At^+^) and/or higher oxidation states and are labile for the electrophilic substitution reactions. The ^211^At species remained in the aqueous solutions for several hours. As described previously, the TLC profile of the aqueous solution of ^211^At indicated the presence of three or more chemical species of ^211^At (Fig. 1). It is speculated that hypervalent species ^211^At^+3^ is also present in the aqueous solution of ^211^At, in addition to ^211^At^+^, ^211^At^0^, and ^211^At^−^^14^. The varying TLC profiles of the aqueous solution of ^211^At obtained from different batches suggest that ^211^At^−^ was easily oxidized by air or oxygen dissolved in the water, affording several higher oxidation states of astatine. Electrophoresis of the aqueous solution of ^211^At revealed that two of the ^211^At species were negatively charged ions while the remaining was a neutral compound; no positively charged species were observed (Supplemental Fig. S5). We hypothesized that they are the ^211^At species with oxidation states of 0, + 1, and/or + 3, which result from the high affinity of these species towards electrophilic substitution reactions. According to the previous reports, the possible chemical forms are At[I]O^−^, At[I](OH)_2_^−^, At[III]O(OH)_2_^−^, At[I]OH, and At[III]O(OH); however, these have not yet been confirmed^15,16^. Unfortunately, it is difficult to determine the chemical structure of the ^211^At species due to the lack of a stable isotope of astatine.

**Figure 6:**
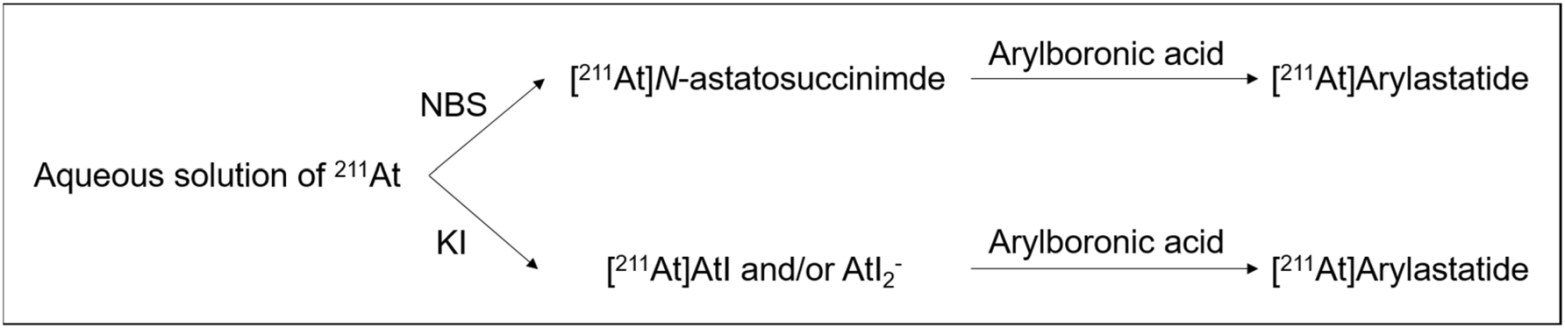
Astatination of arylboronic acids by NBS or KI via the corresponding intermediates.

Based on our findings, the reaction scheme for the ^211^At labeling of arylboronic acids is depicted in Fig. 6. In the NBS method, ^211^At initially reacts with NBS to form the [^211^At]astatosuccinimide intermediate (Fig. 6, top). Then, [^211^At]astatosuccinimide reacts with the arylboronic acid to produce [^211^At]arylastatide. In the KI method, ^211^At reacts with KI to form the [^211^At]AtI and/or AtI2^−^ intermediate (Fig. 6, bottom). Several studies have suggested that astatine can react with iodide and produce inter-halogen compounds such as AtI and AtI_2_^−^^17–19^. The AtI and/or AtI_2_^−^ intermediate reacts with the arylboronic acid to give [^211^At]arylastatide. Compared to NBS, KI was found to be more efficient for the substitution of the dihydroxyboryl group by astatine in the aqueous media. KI can probably reduce the hypervalent ^211^At species present in the aqueous solution (~ 10%), forming the reactive [^211^At]AtI and/or AtI_2_^−^ intermediate that allows higher RCYs compared to that obtained using the NBS method.

^123^I^−^ is relatively stable compared to ^211^At^−^ in aqueous solutions and is not oxidized in air. [^123^I]NaI is usually supplied in a basic aqueous solution to stabilize the iodide ions and prevent the volatilization of hydrogen iodide and iodine. The [^123^I]iodide ion was the sole species in the aqueous solution. BPA was also radiolabeled with ^123^I by the NBS method (Fig. 5, Eq. 2). NBS is known to oxidize iodide ions to the + 1 or zero oxidation state, enabling the iodination of BPA by the dihydroxyboryl-iodine electrophilic substitution reaction. The KI method cannot be employed for the radioiodination of the dihydroxyboryl-substituted compounds because of the lack of any chemical interaction between [^123^I]NaI and KI.

KI was originally intended to be used as the carrier atom of ^211^At since there are no natural stable isotopes of astatine. Unexpectedly, the radiolabeling yield of [^211^At]APA using KI was much higher than 95%, due to the formation of [^211^At]AtI and/or AtI_2_^−^ as a reaction intermediate in the aqueous solution.

It is well known that tyrosine can be labeled with radioactive iodine via an electrophilic substitution reaction in the presence of an oxidant, such as chloramine T, iodogen, *N*-chlorosuccinimde, NBS, or H_2_O_2_. Astatination of tyrosine, however, gave a low yield of [^211^At]astatotyrosine (< 15%) with H_2_O_2_, and the product was unstable under neutral and basic conditions^20,21^. Additionally, the labeling yield was low. These results were suggestive of the different chemical properties of astatine and iodine.

Generally, astatination occurs via electrophilic substitution reactions in the presence of oxidants^22^. In the present system, the astatine species in higher oxidation states are unreactive to the arylboronic acid under the conditions employed, and KI might serve as a reductant to form At^+^. However, we do not have sufficient data at this stage to confirm this possibility.

This methodology has a limitation. As mentioned previously, it is difficult to determine the chemical structures of the ^211^At species, because no authentic compounds are available due to the lack of a natural stable isotope of astatine. Since both iodine and astatine belong to the same group (halogen), we used iodine as the reference and mimetic and compared its behavior with that of astatine.

In summary, we developed a novel method for the synthesis and purification of [^211^At]APA, which has potential clinical uses. The ^211^At labeling of arylboronic acid by the substitution of the dihydroxyboryl group with ^211^At proceeds quickly and generates the product in a high RCY in aqueous media under mild conditions. The advantages and characteristics of our method are as follows. (1) The KI method provides carrier-free product in a high RCY (> 95%) under mild reaction conditions. (2) Purification of the crude reaction mixture in the KI method using SPE provides high recovery yields (> 90%). (3) The entire reaction can be conducted in aqueous media, and no organic solvents are required, except for ethanol in the SPE purification. (4) Only commercially approved drugs and compounds are used for the reaction. (5) No toxic or hazardous reagents are required.

## Methods

### Reagents

BPA purchased from Acros Organics (Morris, US) was dissolved in 7% (w/v) aqueous solution of sodium hydrogen carbonate (Otsuka Pharmaceutical Factory, Tokushima, Japan) at a concentration of 10 mg/mL. NBS, KI, NaI, ascorbic acid, and other reagents were purchased from Nacalai Tesque (Kyoto). NBS, KI, and ascorbic acid were dissolved in WFI (Otsuka Pharmaceutical Factory, JP grade) at concentrations of 4.0 mg/mL, 0.1 mol/L, and 30 mg/mL, respectively. [^123^I]NaI was purchased from Fujifilm Toyama Chemicals (Tokyo, Japan). All solvents used for the experiments were of reagent grade or HPLC grade.

### Thin layer chromatography

The radiolabeled products were analyzed by TLC using a G60 silica gel plate (Merck, Germany). The plate was developed by a 2:1 solvent mixture of acetonitrile (ACN) and water. The TLC plate was exposed to an imaging plate (GE Healthcare, Chicago), and the imaging plate was scanned by Typhoon FLA 7000 (GE Healthcare).

### High-performance liquid chromatography

The radiolabeled products and reference compounds were analyzed by HPLC (LC-20AD, Shimadzu, Kyoto, Japan) using a reversed-phase column (Cosmosil 5C18 MSII, 150 mm × 4.6 mm, Nacalai tesque, Kyoto, Japan). The samples were eluted using a solvent mixture of 20 mmol/L tetrabutylammonium chloride and ACN (7:3) at a flow rate of 1.0 mL/min. The eluate was monitored by a radioactivity flow detector (Gabi star, Elysia-raytest, Belgium) and an UV detector (254 nm).

### Liquid chromatography-mass spectrometry

A Nexera LC–MS system (Shimadzu, Kyoto, Japan) equipped with CROWNPAK CR-I( +) and CR-I(-) columns (3.0 mm i.d., 150 mm, Daicel CPI, Osaka, Japan) was used. A mixture of ACN/water/ethanol/trifluoroacetic acid (80:15:5:0.5) was used as the mobile phase at a flow rate of 0.4 mL/min.

### Production of astatine-211(^211^At)

^211^At was produced by a nuclear reaction of ^209^Bi(α, 2n)^211^At using a cyclotron at the Research Centre for Nuclear Physics at Osaka University (RCNP), RIKEN (Wako, Japan), and the National Institutes for Quantum Radiological Science and Technologies (QST, Chiba and Takasaki, Japan). The produced ^211^At was separated and purified by a dry-distillation method (Fig. S6)^13,23^. The distilled ^211^At was collected in a Teflon tube cooled with ice-water and dissolved in WFI, providing the aqueous solution of ^211^At (10–100 MBq/mL).

### Synthesis of [^211^At]APA

[^211^At]APA was synthesized by the NBS method and KI method.

#### NBS method

One hundred microliters of 1 mg/mL BPA solution containing 7% (w/v) sodium hydrogen carbonate was mixed with 2–80 MBq (5–100 µL) of aqueous solution of ^211^At. Then, 30 µL of 0.4 mg/mL NBS solution was added to the mixture, and the mixture was allowed to stand for 30 min at room temperature. Following this, 100 µL of 30 mg/mL ascorbic acid solution was added to the mixture to stop the reaction.

#### KI method

One hundred microliters of 1 mg/mL BPA solution containing 7% (w/v) sodium hydrogen carbonate was mixed with 2–80 MBq (5–100 µL) of aqueous solution of ^211^At. Then, 50–100 µL of 0.1 mol/L KI solution was added to the mixture, and the mixture was allowed to stand for 30 min at room temperature.

### Purification of [^211^At]APA

The crude reaction mixture of [^211^At]APA was purified by SPE. The mixture was loaded onto an Oasis HLB cartridge (Waters, Milford, US), and the cartridge was rinsed with 1 mL of WFI. [^211^At]APA trapped in the cartridge was eluted by 1 mL of 30% (v/v) ethanol.

### Synthesis of [^123^I]IPA and non-radioactive IPA

Iodine-123-labeled 4-iodo-L-phenylalanine ([^123^I]IPA) was synthesized using iodine-123, ([^123^I]NaI, 74MBq) and BPA in a manner same as that for the synthesis of [^211^At]APA. Separately, sodium iodide containing the stable isotope of iodine was reacted with chemically equivalent amounts of BPA and NBS in order to elucidate the structure of the product. The resulting product and the authentic reference, IPA, were analyzed using LC–MS.

## Supplementary Information

Supplementary Information.
