# Supplementary material for: Synthesis of [211At]4-astato-L-phenylalanine by dihydroxyboryl-astatine substitution reaction in aqueous solution
Source: Sci Rep. 2021 Jun 21;11:12982. doi: 10.1038/s41598-021-92476-6 (PMC8217504; doi:10.1038/s41598-021-92476-6)
Supplement: Supplementary file 1 — Supplementary Information. [file 41598_2021_92476_MOESM1_ESM.pdf]

Synthesis of [^{211}At]4-astato-L-phenylalanine by dihydroxyboryl-astatine substitution reaction in aqueous solution

Yoshifumi Shirakami,[1,2*] Tadashi Watabe,[1,3] Honoka Obata,[4] Kazuko Kaneda,[1] Kazuhiro Ooe,[1,3] Yuwei Liu,[3] Takahiro Teramoto,[1] Atsushi Toyoshima,[1] Atsushi Shinohara,[1,3] Eku Shimosegawa,[5] Jun Hatazawa,[2] and Koichi Fukase[1,4]

FIGURES

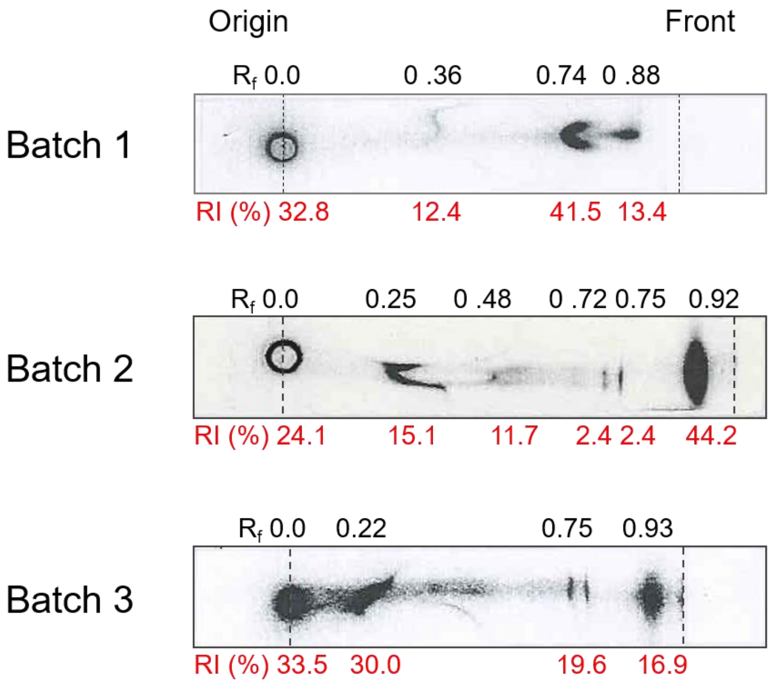

Fig. S1 Thin layer chromatogram of aqueous solution of ^{211}At

The radioactivity profiles of the chromatograms varied from batch to batch.

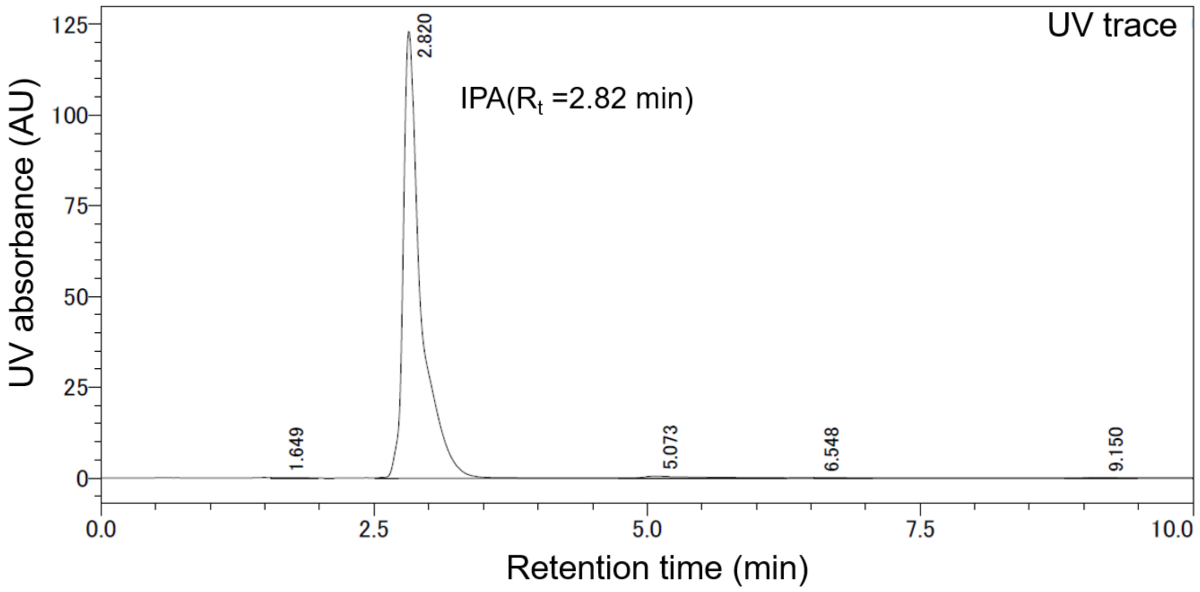

Fig. S2 HPLC profiles of 4-iodo-L-phenylalanine (IPA)

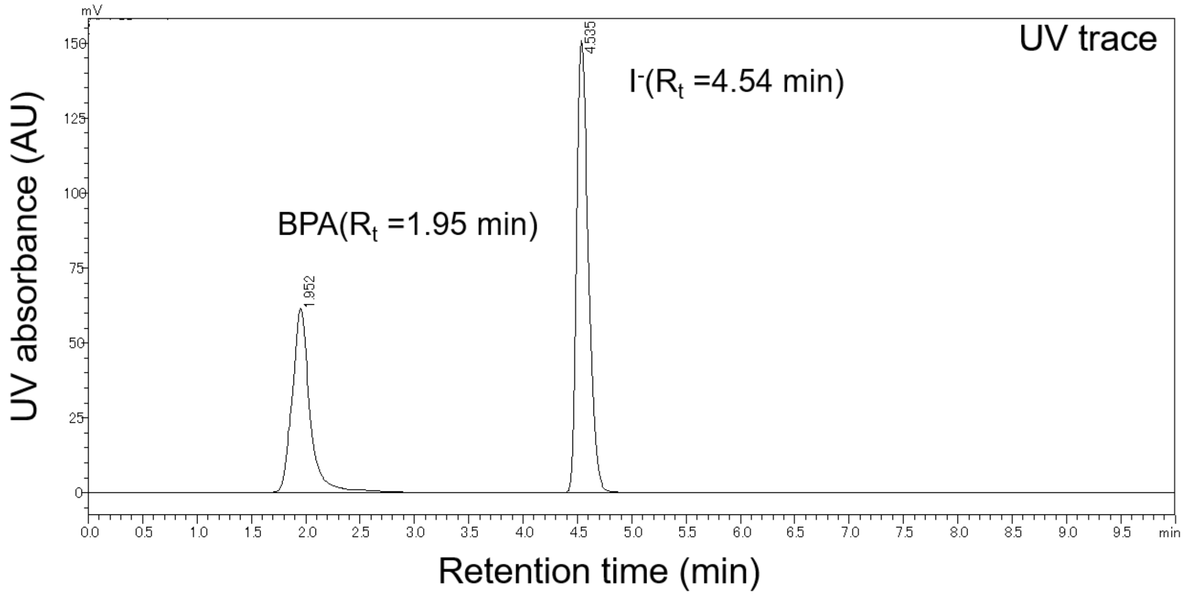

Fig. S3 HPLC profile of 1:1 mixture of BPA and KI

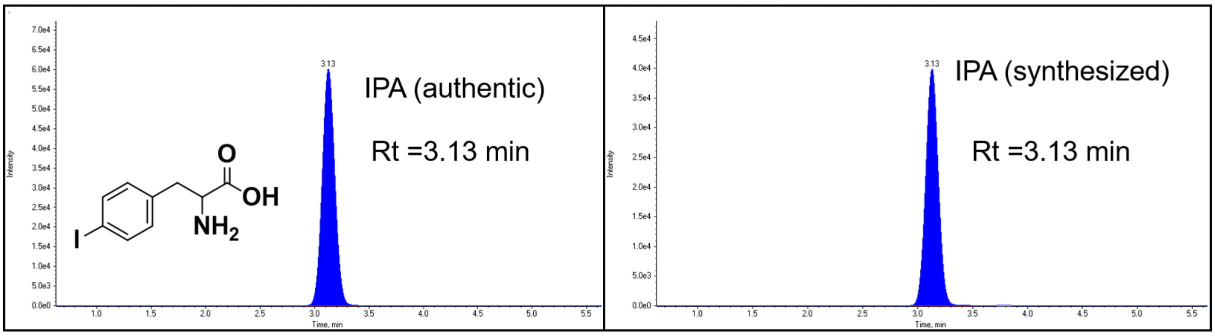

Fig. S4 LC-MS analysis of IPA

Authentic reference (left) and synthesized product from the boron-iodine substitution reaction between BPA and NaI (right).

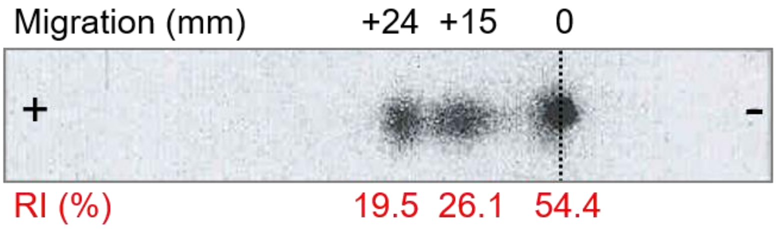

Fig. S5. Electrophoresis of aqueous solution of ^{211}At

Strip: Cellulose acetate film, Electrolyte: 0.06 mol/L barbital buffer, pH 8.5,
Constant voltage: 133 V, Constant current: 1 mA/cm, Time duration: 20 min

Three radioactive species were detected in the aqueous solution of ^{211}At. Two of them were negatively charged ions having migration distances of +15 mm and +24 mm. The third species was neutral and remained on the origin line (0 mm).

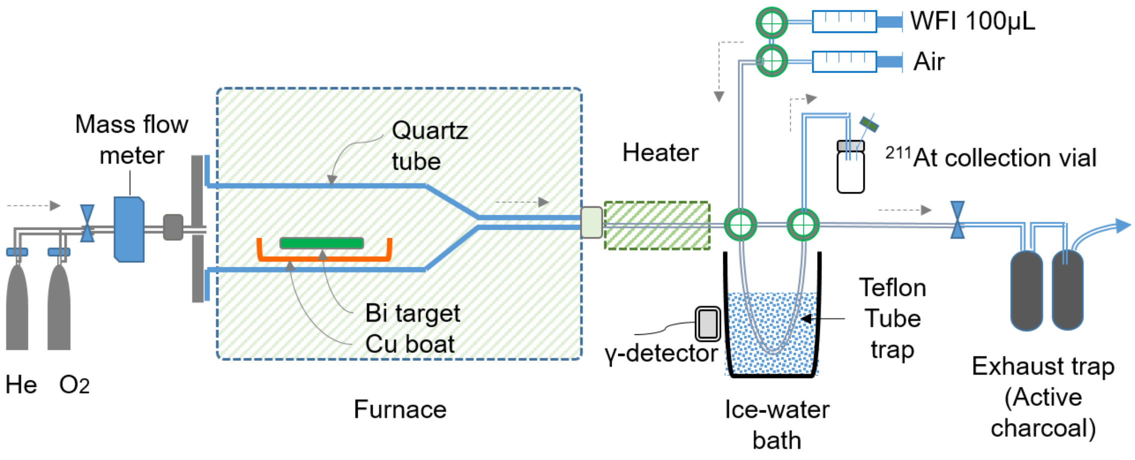

Fig. S6 Apparatus for dry-distillation of ^{211}At
